# Association of postprandial postexercise muscle protein synthesis rates with dietary leucine: A systematic review

**DOI:** 10.14814/phy2.15775

**Published:** 2023-08-03

**Authors:** Kiera Wilkinson, Christopher P. Koscien, Alistair J. Monteyne, Benjamin T. Wall, Francis B. Stephens

**Affiliations:** ^1^ Nutritional Physiology Research Group, Public Health and Sport Sciences, Faculty of Health and Life Sciences University of Exeter Exeter UK

**Keywords:** amino acids, exercise, leucine, muscle protein synthesis, protein

## Abstract

**Background:**

Dietary protein ingestion augments post (resistance) exercise muscle protein synthesis (MPS) rates. It is thought that the dose of leucine ingested within the protein (leucine threshold hypothesis) and the subsequent plasma leucine variables (leucine trigger hypothesis; peak magnitude, rate of rise, and total availability) determine the magnitude of the postprandial postexercise MPS response.

**Methods:**

A quantitative systematic review was performed extracting data from studies that recruited healthy adults, applied a bout of resistance exercise, ingested a bolus of protein within an hour of exercise, and measured plasma leucine concentrations and MPS rates (delta change from basal).

**Results:**

Ingested leucine dose was associated with the magnitude of the MPS response in older, but not younger, adults over acute (0–2 h, *r* ^2^ = 0.64, *p* = 0.02) and the entire postprandial (>2 h, *r* ^2^ = 0.18, *p* = 0.01) period. However, no single plasma leucine variable possessed substantial predictive capacity over the magnitude of MPS rates in younger or older adults.

**Conclusion:**

Our data provide support that leucine dose provides predictive capacity over postprandial postexercise MPS responses in older adults. However, no threshold in older adults and no plasma leucine variable was correlated with the magnitude of the postexercise anabolic response.

## 1 INTRODUCTION

Prolonged resistance exercise training increases skeletal muscle mass and strength, advantageous adaptive responses to support athletic/health goals in a range of individuals (Damas et al., 2016; Farup et al., 2012). Training‐induced increases in muscle mass are mechanistically underpinned by persistent periods of positive net protein balance, and therefore protein accretion, within muscle tissue (Fujita et al., 2007). A single bout of resistance exercise stimulates muscle protein synthesis (MPS) rates, peaking in the immediate hours subsequent (~2–6 h; Moore, Tang, et al., 2009; van Vliet et al., 2019) and remaining elevated for ~24–48 h (Biolo et al., 1995; Burd et al., 2011; Phillips et al., 1997). However, resistance exercise also stimulates muscle protein breakdown (MPB) rates such that, in the postabsorptive state, muscle protein net balance remains negative (Biolo et al., 1995). As a result, nutrition plays a vital role in promoting postexercise net positive protein balance in muscle and, therefore, muscle reconditioning.

Protein ingestion before (Burke et al., 2012; Tipton et al., 2007), immediately after (Brook et al., 2021; Moore, Robinson, et al., 2009; Pennings, Koopman, et al., 2011; Tang et al., 2009), and up to 24‐h (Elliot et al., 2006; Holwerda et al., 2016; Kim et al., 2016) postresistance exercise augments the rise in MPS rates and, albeit less potently, inhibits MPB rates (Biolo et al., 1997) resulting in a positive net muscle protein balance. The magnitude of the (postexercise) MPS response to protein ingestion appears to increase in a dose–response manner plateauing somewhere between 20 and 40 g (Moore, Robinson, et al., 2009; Witard et al., 2014; Yang et al., 2012), a relationship that shifts to the right in more anabolically insensitive older adults (Burd et al., 2013; Cuthbertson et al., 2005; Rennie & Wilkes, 2005; Wall, Gorissen, et al., 2015). However, as opposed to total protein per se, this relationship has been suggested to be more contingent on essential amino acids (Bohé et al., 2003; Cuthbertson et al., 2005; Fujita et al., 2007; Tipton et al., 1999) and, in particular, leucine (Phillips, 2016; Rieu et al., 2006), which has a well‐characterized molecular role in stimulating the mTORC1/P70S6K signaling pathway (the major myocellular anabolic cascade; Dreyer et al., 2008; Drummond & Rasmussen, 2008; Layman, 2002; Norton & Layman, 2006). However, the nature of the relationship between leucine and postprandial MPS (particularly when considered in the postexercise phase) remains to be fully defined.

Some reports imply that simply the amount of leucine contained within the ingested food/meal directly dictates postprandial MPS rates (i.e., “leucine threshold” concept; Breen & Phillips, 2011). This pragmatic dose–response view allows simple recommendations to be made, such as ~2 to ~3 g to be consumed for measurable and optimal postexercise MPS responses, respectively (Phillips et al., 1997; Volpi et al., 2013). Other reports take account of physiological variables such as protein digestion and amino acid absorption which, together, dictate peripheral leucine availability following protein ingestion and, thus, a stimulus actually seen by the muscle (i.e., “leucine trigger” concept; Tang et al., 2009; West et al., 2011). However, even within this more sophisticated view, it is unclear whether the peak concentration (Tang et al., 2009; West et al., 2011), rate of rise (Phillips & Van Loon, 2011; West et al., 2011), or total postprandial availability of plasma (Oikawa et al., 2020; or even intramuscular unbound) leucine is the prime “trigger.” A recent qualitative systematic review (Zaromskyte et al., 2021) supported the utility of the leucine trigger hypothesis within muscle of older individuals and during studies where crystalline amino acid mixtures or isolated proteins were ingested. The predictive value of the hypothesis diminished in younger subjects and/or where protein‐rich whole foods (within their unique matrices; Beals et al., 2018; Burd et al., 2015; Elliot et al., 2006; Van Vliet et al., 2017) and/or mixed meals (Kim et al., 2016; Symons et al., 2011) were ingested. However, the definition of the leucine trigger in this review was binary and defined only as a “greater overall plasma leucine response,” which did not allow for any quantitative dose–response relationship to be established.

We conducted a quantitative systematic review with the primary aim of refining our understanding of the relationship between ingested leucine and the magnitude of postprandial postexercise MPS rates. We compiled data from human studies that applied a study design including bolus ingestion of amino acids or protein (either alone or contained within a food/meal) and the execution of a single resistance exercise bout, combined with the parallel measurements of MPS rates, and with further inclusion criteria applied around postprandial plasma leucine concentrations. We clearly demarcated between the leucine threshold hypothesis (i.e., leucine dose), and the three distinct (sub)variables identified within the leucine trigger hypothesis (i.e., peak magnitude, rate of rise, and total availability of plasma leucine) and evaluated their relationships with the magnitude of postprandial postexercise MPS responses in young and older adults.

## 2 METHODS

This study was registered on, and the protocol was uploaded to, PROSPERO (CRD42021227295). The review was conducted based on PRISMA guidelines 2020, in line with quantitative systematic reviews (Moher et al., 2009). The primary outcome of this systematic review was to refine our understanding of the relationship between ingested leucine and the magnitude of postprandial postexercise MPS rates. Heterogeneity in the absolute determination of MPS rates and plasma leucine concentrations between studies, methods, and laboratories was accounted for by assessing delta changes on both variables (MPS and plasma leucine concentrations) as our primary depiction of the data.

### 2.1 Search strategy

A systematic search of the literature was conducted in Medline (https://pubmed.ncbi.nlm.nih.gov/), Cochrane (https://www.cochranelibrary.com/central), and Embase (https://www.embase.com/) databases on the July 12, 2022. The medical subject headings (MeSH) “Leucine”, “Protein Biosynthesis”, “Dietary Supplements”, “Dietary Proteins” and “Muscle Proteins” were utilized. Boolean operators “AND” and “OR” were used to combine search terms. The following search terms were used (protein OR leucine OR amino acid OR supplement* OR diet* OR consume* OR intake* OR ingest* OR powder OR drink* OR shake OR isolate) AND (muscle* OR myofibrillar* OR mixed OR muscular OR protein synthesis) AND (weight* OR resistance* OR strength OR isometric OR train* OR exercise OR lift*) AND (randomized OR randomized control trial).

### 2.2 Eligibility criteria

All randomized controlled trials (RCT) reporting MPS rates in healthy human adults after bolus ingestion of an amino acid, amino acid mixture, isolated protein source, protein containing whole food, or mixed meal in close temporal proximity (maximally 1 h before or after) to a bout of resistance exercise (exercise against an external load) were considered for inclusion. Resistance exercise protocols were considered broadly, with a variety of exercise protocols included, differing in modalities and mechanics of movement. All these protocols were designed and considered to provide a maximal stimulus, and therefore, we assume that this broad study inclusion allowed the examination of a “maximal” exercise‐induced stimulation of MPS and the further examination of how this is modulated by leucine consumption.

Further inclusion was applied whereby studies had to report plasma leucine concentrations for at least 1.5 h after protein ingestion with time intervals of, at most, 30 min to calculate peak plasma leucine magnitude, rate of rise to peak plasma leucine magnitude, and total postprandial plasma leucine availability. MPS rates needed to be determined by the primed continuous infusion of stable isotopically labeled amino acid(s) (though specific isotope or in which amino acid it was labeled was not required) and plasma leucine concentrations via venous or arterialized‐venous blood sampling methods, with quantification of amino acid concentrations via gas chromatography–mass spectrometry (GC–MS).

### 2.3 Exclusion criteria

Studies that were excluded were as follows: those where participants were classified as unhealthy; if a source of protein was provided via intravenous infusion or repeated doses (as opposed to a single bolus); if more than one resistance exercise session was performed; where the protein bolus was provided more than 1‐h pre or postexercise; or, if an acute measurement period of MPS was not available.

### 2.4 Data collection

Two reviewers (K.W. and C.P.K.) screened all titles and abstracts to identify potentially eligible studies, and full papers were obtained and assessed for inclusion independently by these authors. Any disagreement regarding eligibility was resolved through deliberation or referred to a third‐party author (A.J.M.), to resolve the decision, if necessary. All duplicates were identified and removed. Automation tools within the Rayyan software (https://www.rayyan.ai/) were used to filter key words, to detect studies that did not fit the inclusion criteria (i.e., study populations in rats, pigs, and children).

### 2.5 Data extraction

Predetermined relevant outcome variables from each study were extracted by one reviewer (K.W.) and the other reviewer (C.P.K.) revisited all to check for discrepancies. Relevant variables included: number of participants, participant characteristics (age, sex, and training status, if supplied), protein supplementation protocol (protein dose and leucine dose), exercise intervention, mixed muscle or myofibrillar protein synthesis values and plasma leucine concentrations. If protein or leucine doses were given relative to body weight, this was calculated with the mean body weight of the participants in that group to normalize all data to absolute doses. If the study in question did not provide the leucine dose within the nutritional content provided for the protein source, then manufacturer information was searched (Burd, Andrews, et al., 2012; Burd, Yang, et al., 2012; Chan et al., 2019; Dideriksen et al., 2016; McKendry et al., 2016; Mikkelsen et al., 2015) or corresponding authors were contacted to provide manufacturer information (Agergaard et al., 2017; Areta et al., 2014; McGlory et al., 2016) or nutritional analysis (Holwerda et al., 2016). If the study included more than one protein source (e.g., whey and soy), outcome measures were taken for both and treated as separate study arms. If the study included a placebo or control group that was not a protein source, this was not included in data extraction. Protein sources with additional fortification and co‐ingestion with macronutrients were noted and included. Data were further characterized into whole food sources (mixed macronutrient nonsupplemental protein) and nonwhole food sources. This is represented visually within the presented dataset herein. Data were considered as a whole dataset and then organized into young and older participants. This was based on the descriptive statistics of the participants provided. The mean age of the younger participants within a given study was required to be between 18 and 40 years. The mean age of the older participants within a given study was required to be >55 years. This was to ensure that the threshold for the onset of age‐related sarcopenia had been met (Janssen, 2010). Where numeric data were not reported in tables or text, and authors could not be reached, data were extracted from charts and figures using Web Plot Digitizer (https://automeris.io/WebPlotDigitizer/).

### 2.6 Risk of bias

The Cochrane Handbook and tools (Higgins et al., 2011, 2019) were used for the risk of bias assessment for each individual study. The quality of each study was assessed by one reviewer (K.W.) and checked by another reviewer (C.K.), and any disagreement was resolved through deliberation between K.W. and C.K. Six main criteria were assessed, and the quality of each study was based on high, low or unclear risk of bias (Supplementary Information—https://doi.org/10.6084/m9.figshare.22203514). Studies with a high risk of bias were due to blinding procedures, usually in the case of whole food protein sources (Beals et al., 2018; Symons et al., 2011), where blinding of the allocated intervention was not possible. Sequence generation was considered a high risk of bias when allocation to the intervention was based on a criterion such as younger or older participants; therefore, assignment to the intervention was nonrandom. Both high risk of bias variables were considered satisfactory for this dataset.

### 2.7 Synthesis methods

The main outcome variables used in this review have been converted from the data extracted and have been used to visually display the data in the figures. Basal [postabsorptive] fractional synthetic rate (FSR) and postexercise postprandial FSR (%/h) were used to calculate delta change (ΔFSR [%]) for normalization across studies. These were then split into early (0–2‐h postexercise and/or protein ingestion) and the entire postprandial (0–6‐h postexercise and/or protein ingestion) MPS response. Plasma leucine concentrations were displayed as peak plasma leucine concentration (highest single mean value reported), rate of rise to peak plasma leucine magnitude (peak plasma leucine concentration minus basal plasma leucine concentration divided by time in minutes to peak plasma leucine concentration), and total postprandial plasma leucine availability (incremental area under the curve [iAUC/180 min]). Data were analyzed using linear regression; coefficient of determination (*r* ^2^), significance (*p* value), and *y*‐intercept (*b*_0_) have been presented for interpretation. Subject characteristics are presented as mean ± SD.

## 3 RESULTS

### 3.1 Literature search and study inclusion

**FIGURE 1:**
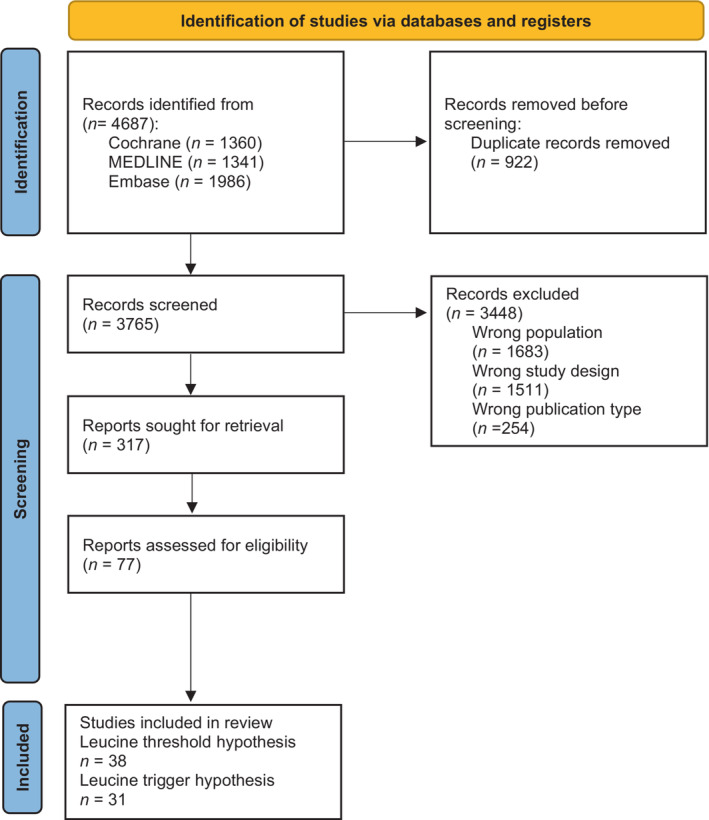
Flow diagram of the screening process in accordance with the PRISMA guidelines.

Figure 1 shows the process of article selection with 38 studies ultimately included. Within these 38 studies, there were 77 study arms (i.e., total number of eligible intervention groups), to determine aspects relating to the leucine threshold hypothesis (Supplementary Information—https://doi.org/10.6084/m9.figshare.22203514, Agergaard et al., 2017; Areta et al., 2014; Atherton et al., 2017; Beals et al., 2018; Borack et al., 2016; Brook et al., 2021; Bukhari et al., 2015; Burd et al., 2010, 2015; Burd, Andrews, et al., 2012; Burd, Yang, et al., 2012; Chan et al., 2019; Churchward‐Venne, Breen, et al., 2014; Churchward‐Venne, Cotie, et al., 2014; Devries et al., 2018a, 2018b; Dickinson et al., 2014; Dideriksen et al., 2016; Dreyer et al., 2008; Fujita et al., 2009; Gwin et al., 2021; Hermans et al., 2021, 2022; Luiking et al., 2014; McGlory et al., 2016; McKendry et al., 2016; Mikkelsen et al., 2015; Monteyne, Coelho, Porter, Abdelrahman, Jameson, Finnigan, et al., 2020; Monteyne, Coelho, Porter, Abdelrahman, Jameson, Jackman, et al., 2020; Moore, Tang, et al., 2009; Oikawa et al., 2020; Pinckaers et al., 2022; Reidy et al., 2013; Reitelseder et al., 2019; Symons et al., 2011; Van Vliet et al., 2017; West et al., 2009; Wilkinson et al., 2018). Studies which met all the inclusion criteria except not taking a basal muscle biopsy (i.e., Dideriksen et al., 2011) were excluded in order to calculate delta change from basal MPS.

Of these 38 identified RCTs, six studies were crossover in design (Areta et al., 2014; Brook et al., 2021; Burd et al., 2015; Gwin et al., 2021; Van Vliet et al., 2017; West et al., 2009), whereas the remainder were parallel design. Double‐blinding procedures were used in 11 of the 38 studies, five were single‐blinded, and the remainder were unblinded. Further exclusion was applied, whereby seven studies did not meet the criteria of providing plasma leucine concentrations, leaving 31 studies (61 study arms) included in the further analysis to determine aspects of the leucine trigger hypothesis (Supplementary Information—https://doi.org/10.6084/m9.figshare.22203514).

### 3.2 Participants' characteristics

Of the total 38 studies, five of the studies recruited females only (Bukhari et al., 2015; Devries et al., 2018a, 2018b; Oikawa et al., 2020; Wilkinson et al., 2018) and six recruited males and females (Areta et al., 2014; Beals et al., 2018; Fujita et al., 2009; Luiking et al., 2014; Reidy et al., 2013; Symons et al., 2011), with the remainder in males only. Of the 77 study arms relating to the leucine threshold analysis, 45 studies were of younger adults and 32 of older adults. The age range of younger participants was 19–29 years (23 ± 2.3 years, 409 male and 40 female participants), and the age range of older adults was 57–74 years (68 ± 3.5 years, 142 male and 106 female participants). Of the 61 study arms relating to the leucine trigger analysis, 35 were studies of younger adults and 26 of older adults. Eleven study arms defined their participants as resistance‐trained individuals, while 19 study arms stated the participants were recreationally active. Furthermore, six study arms recruited low‐to‐moderately active participants with the remainder of the study arms reporting healthy individuals with no specific training status provided.

### 3.3 Protein sources

Bolus doses of orally administered isolated proteins comprised: whey (33 study arms), casein (three study arms), milk protein concentrate (10 study arms), crystalline essential amino acid mixtures (nine study arms), and isolated protein blends (four study arms). Other sources included protein‐rich foods: pork (two study arms; Beals et al., 2018), beef (three study arms; Burd et al., 2015; Symons et al., 2011), mycoprotein (two study arms; Monteyne, Coelho, Porter, Abdelrahman, Jameson, Finnigan, et al., 2020; Monteyne, Coelho, Porter, Abdelrahman, Jameson, Jackman, et al., 2020), protein‐rich meal replacements (four study arms; Atherton et al., 2017), cheese (Hermans et al., 2022), meal worms (Hermans et al., 2021), collagen protein, potato protein (Pinckaers et al., 2022), egg white (Van Vliet et al., 2017), and egg yolk (Van Vliet et al., 2017), all with one study arm each.

### 3.4 Resistance exercise interventions

A unilateral exercise model was used for 27 out of the 38 studies, with the remaining 11 studies using a bilateral exercise model. The exercise protocols consisted of a variety of reps and sets ranging from 1 to 10 sets and from 8 to 36 reps or to volitional exhaustion/failure. All studies had a familiarization with the exercise equipment and tested for maximum strength to determine the workload. The intensity ranged from 16% to 90% of one repetition maximum. Within three study arms, maximal leg extension exercise was on a dynamometer (Monteyne, Coelho, Porter, Abdelrahman, Jameson, Finnigan, et al., 2020; Monteyne, Coelho, Porter, Abdelrahman, Jameson, Jackman, et al., 2020). The exercise protocol was either leg press (one study arm; Areta et al., 2014), leg/knee extension (54 study arms), both leg press and leg extension (20 study arms), arm cable curl (elbow flexion; one study arm; West et al., 2009), or combination of upper and lower body resistance exercise session (one study arms; West et al., 2009).

### 3.5 Postprandial postexercise periods

The measurement of MPS was taken within the mixed muscle protein fraction for four studies (Gwin et al., 2021; Hermans et al., 2021; Monteyne, Coelho, Porter, Abdelrahman, Jameson, Finnigan, et al., 2020; Monteyne, Coelho, Porter, Abdelrahman, Jameson, Jackman, et al., 2020) and the remaining studies measured MPS in myofibrillar proteins. The basal biopsy was conducted preintervention, and the incorporation period for the measurement of MPS ranged from 1.5‐ to 6‐h postprandial, postexercise. All studies collected muscle biopsy tissue from the *m. vastus lateralis*, except one study with measurements of MPS from tissue collected from the *biceps brachii* (West et al., 2009).

### 3.6 Leucine dose

**FIGURE 2:**
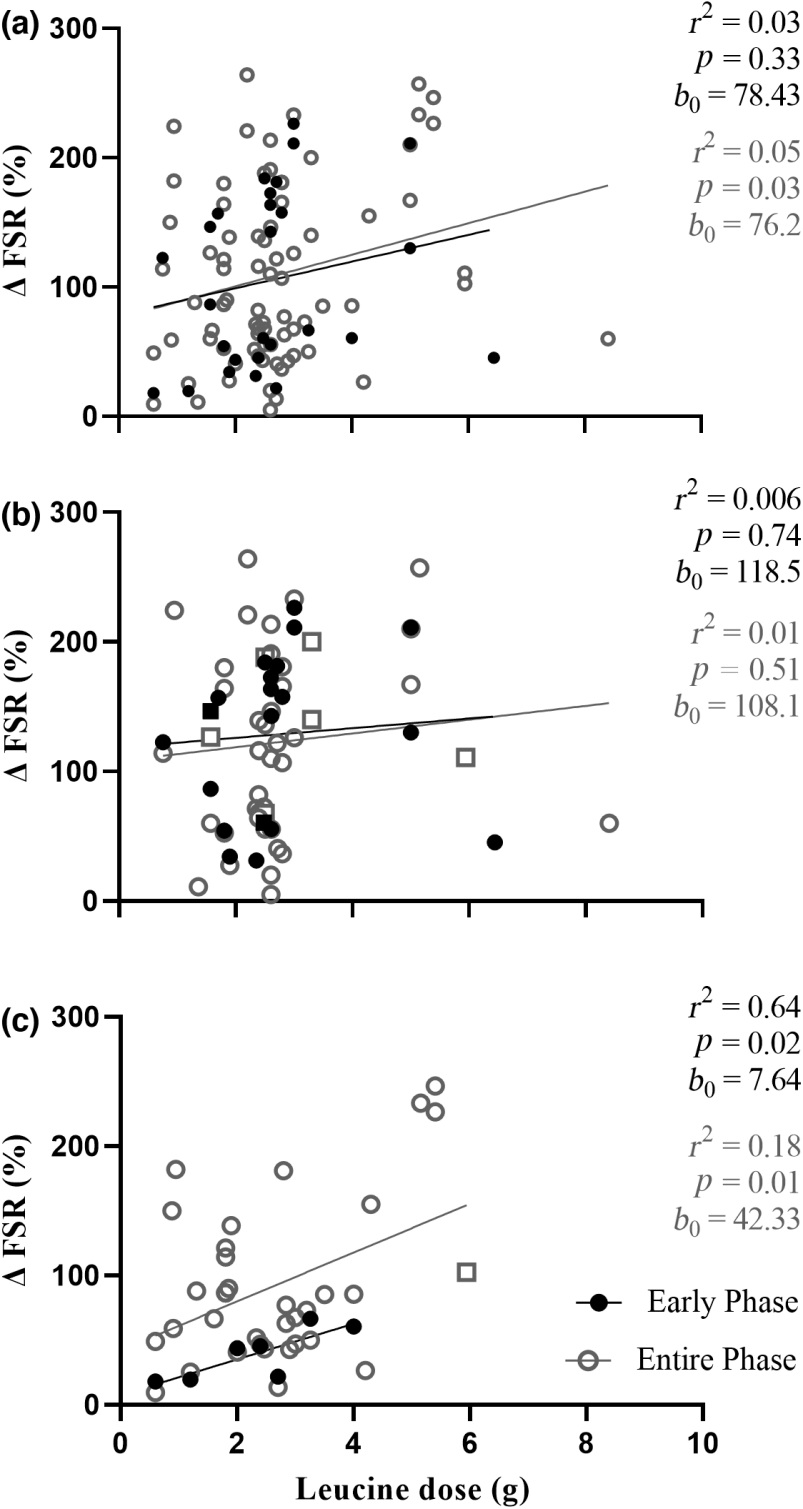
Delta change (postprandial postexercise increase) in muscle protein synthesis rates (MPS), early (0–2 h) and during the entirety of the postprandial period, expressed as fractional synthetic rate (FSR), in response to a leucine dose in all participants (77 study arms) (a), young participants (45 study arms; 19–29 years) (b) and older participants (32 study arms; 57–74 years) (c). Squares represent whole food sources. Data were analyzed by linear regression; coefficient of determination (*r* ^2^), *p* value, and *y*‐intercept (*b*_0_) are presented.

A graphical depiction of the relationship between ingested leucine dose and the delta change in postprandial, postexercise MPS rates is represented in Figure 2, with data illustrated as an early (0–2 h) and entire measurement (>2 h) phase, and presented as an entire dataset (A), and for young (B) and older (C) adults separately. When considering the entire dataset (Figure 2a), leucine dose showed no relationship to delta change in postexercise MPS rates over the early phase (*r* ^2^ = 0.03, *p* = 0.33, *b*_0_ = 78.43), but a significant correlation was observed over the entire measurement period (*r* ^2^ = 0.05, *p* = 0.03, *b*_0_ = 76.2). The latter was mainly driven by data obtained from older adults, given divergent responses between the age categories were observed. Specifically, the relationship between leucine dose and postprandial postexercise MPS change was not present in young adults (Figure 2b) over either the early (*r* ^2^ = 0.006, *p* = 0.74, *b*_0_ = 118.5) or entire (*r* ^2^ = 0.01, *p* = 0.51, *b*_0_ = 108.1) measurement periods, whereas correlations were observed over both periods (*r* ^2^ = 0.64, *p* = 0.02, *b*_0_ = 7.64 and *r* ^2^ = 0.18, *p* = 0.01, *b*_0_ = 42.33 over the early and entire measurements periods, respectively) in older adults (Figure 2c).

### 3.7 Leucine trigger hypothesis

**FIGURE 3:**
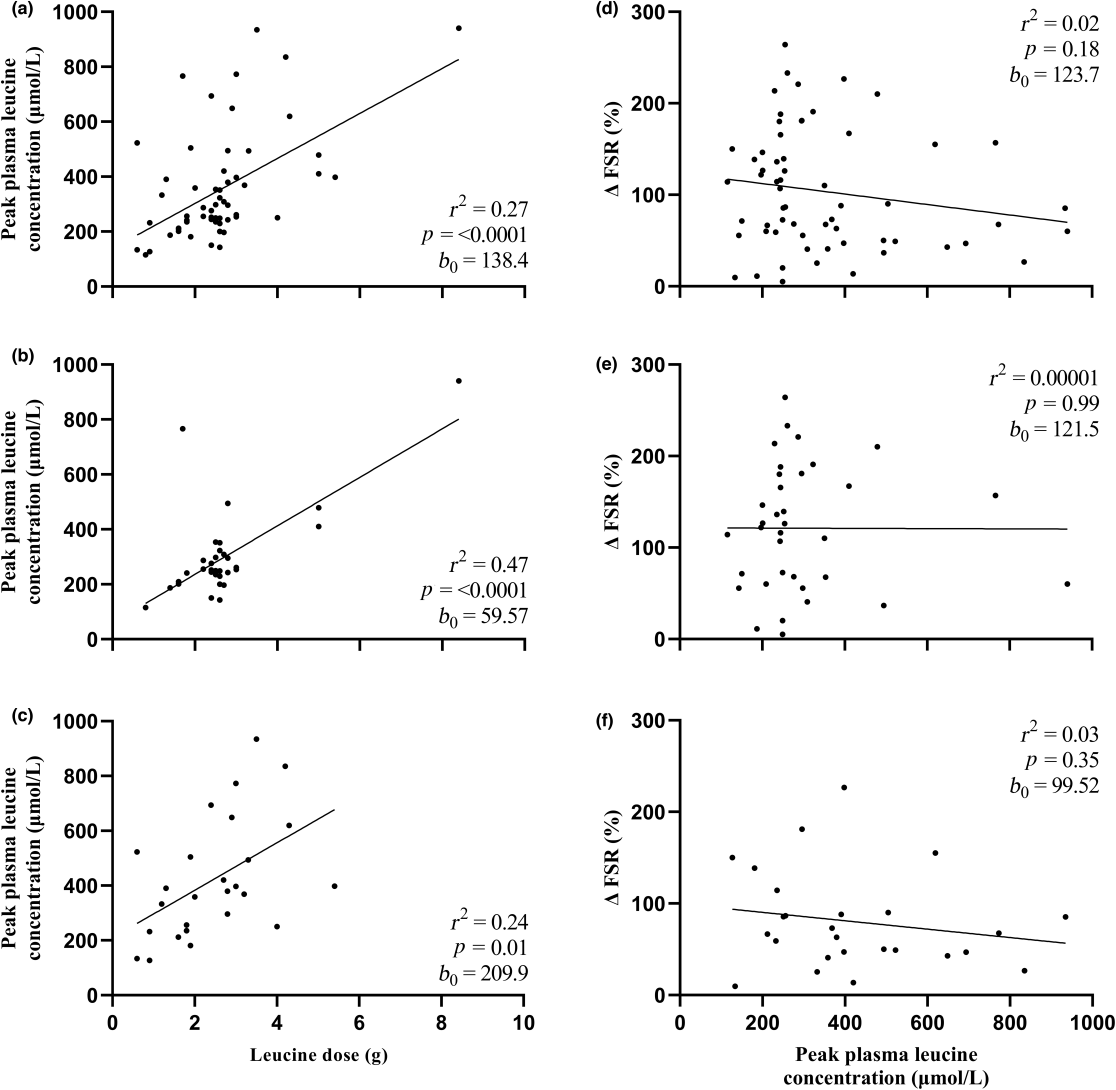
Peak plasma leucine magnitude (highest mean value reported) in response to a leucine dose provided as a bolus protein ingestion within an hour of resistance exercise in all participants (61 study arms) (a), young participants (35 study arms; 19–29 years) (b) and older participants (26 study arms; 57–74 years) (c). Delta change (postprandial postexercise increase) in muscle protein synthesis rates (MPS), expressed as fractional synthetic rate (FSR) in relation to peak plasma leucine magnitude in all participants (d), young participants (e) and older participants (f). Data were analyzed by linear regression; coefficient of determination (*r* ^2^), *p* value, and *y*‐intercept (*b*_0_) are presented.

**FIGURE 4:**
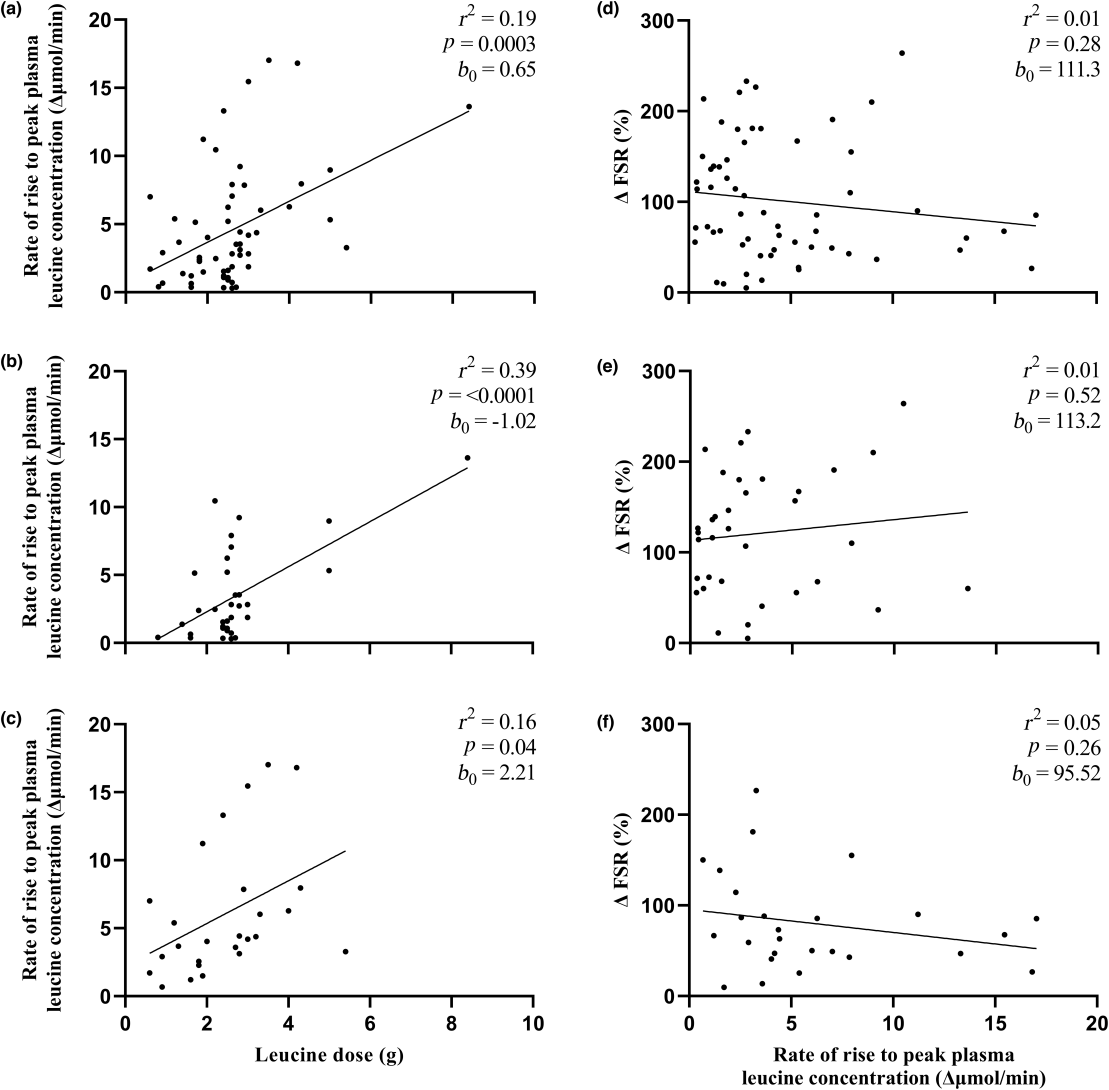
Rate of rise to peak plasma leucine magnitude in response to a leucine dose provided as a bolus protein ingestion within an hour of resistance exercise in all participants (61 study arms) (a), young participants (35 study arms; 19–29 years) (b) and older participants (26 study arms; 57–74 years) (c). Delta change (postprandial postexercise increase) in muscle protein synthesis rates (MPS), expressed as fractional synthetic rate (FSR) in relation to rate of rise to peak plasma leucine magnitude in all participants (d), young participants (e) and older participants (f). Rate of rise to peak plasma leucine magnitude determined from the highest plasma leucine concentration minus basal plasma leucine concentration divided by time in minutes to peak concentration. Data were analyzed by linear regression; coefficient of determination (*r* ^2^), *p* value, and *y*‐intercept (*b*_0_) are presented.

**FIGURE 5:**
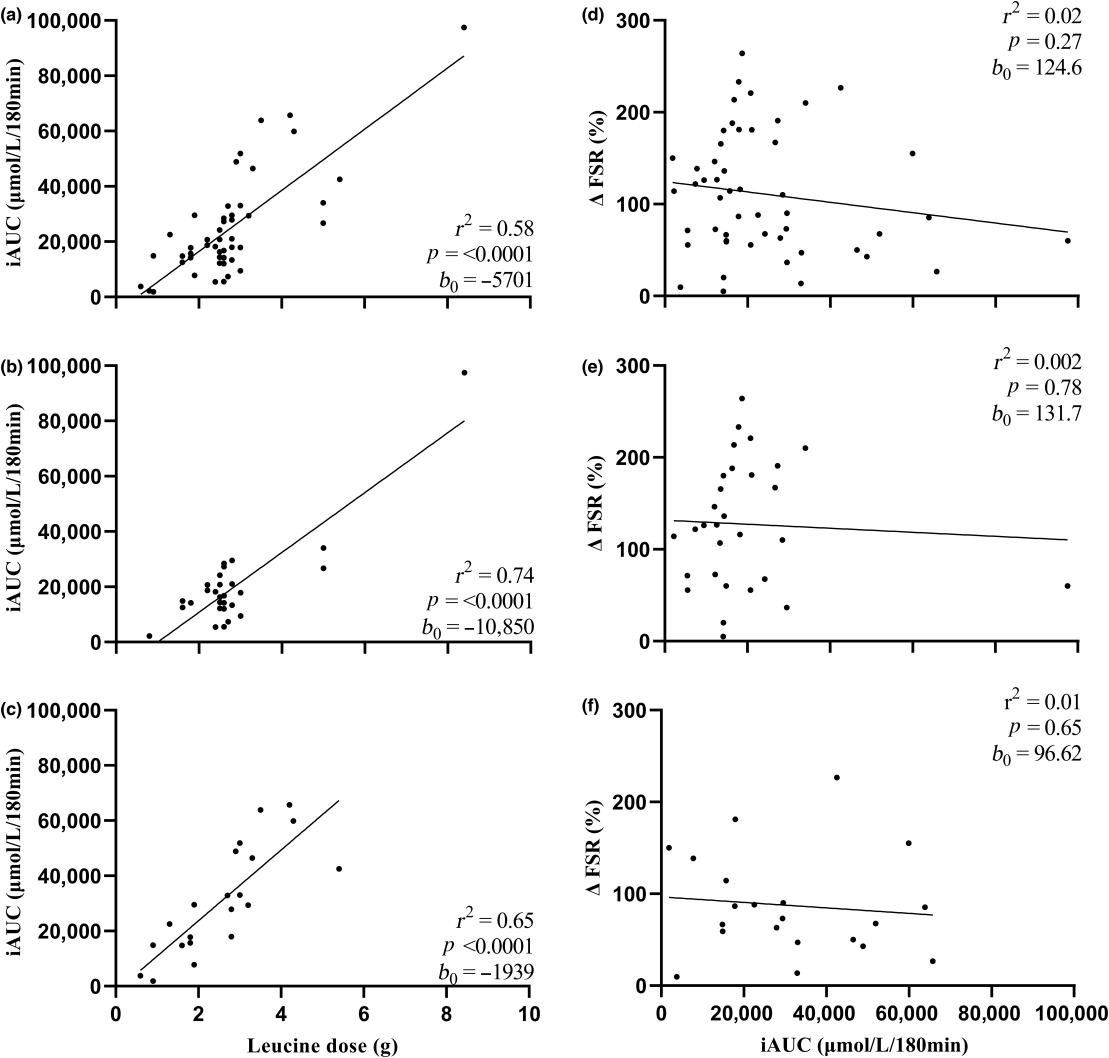
Total postprandial plasma leucine availability, represented as incremental area under the curve over 180 min, in response to a leucine dose provided as a bolus protein ingestion within an hour of resistance exercise in all participants (61 study arms) (a), young participants (35 study arms; 19–29 years) (b) and older participants (26 study arms; 57–74 years) (c). Delta change (postprandial postexercise increase) in muscle protein synthesis rates (MPS), expressed as fractional synthetic rate (FSR) in relation to total postprandial leucine availability in all participants (d), young participants (e) and older participants (f). Data were analyzed by linear regression; coefficient of determination (*r* ^2^), *p* value, and *y*‐intercept (*b*_0_) are presented.

A graphical depiction of the relationship between ingested leucine dose and the peak magnitude, rate of rise, and total postprandial availability of plasma leucine are represented in Figures 3, 4, 5, respectively, with data presented as an entire dataset (A), and for young (B) and older (C) adults separately. Further graphical depiction of the relationship between delta change in postprandial, postexercise MPS and peak magnitude, rate of rise, and total postprandial availability of plasma leucine are represented as an entire dataset (D), and for young (E) and older (F) adults separately.

#### 3.7.1 Peak plasma leucine magnitude

When considering the entire dataset (Figure 3a), the leucine dose showed a relationship with peak plasma leucine magnitude (*r* ^2^ = 0.27, *p* < 0.0001, *b*_0_ = 138.4). A significant relationship was observed between leucine dose and peak plasma leucine magnitude for both younger (Figure 3b; *r* ^2^ = 0.47, *p* < 0.0001, *b*_0_ = 59.57) and older (Figure 3c; *r* ^2^ = 0.24, *p* = 0.01, *b*_0_ = 209.9) adults. However, peak plasma leucine magnitude showed no relationship to delta change in postprandial, postexercise MPS rates when considering the whole dataset (Figure 3d; *r* ^2^ = 0.02, *p* = 0.18, *b*_0_ = 123.7) and this relationship was not altered when split for age; leucine dose showed no relationship to delta change in postexercise MPS rates among the younger (Figure 3e; *r* ^2^ = 0.00001, *p* = 0.99, *b*_0_ = 121.5) or older (Figure 3f; *r* ^2^ = 0.03, *p* = 0.35, *b*_0_ = 99.52) adults.

#### 3.7.2 Rate of rise to peak plasma leucine magnitude

When considering the whole dataset (Figure 4a), there was a relationship between leucine dose and rate of rise to peak plasma leucine magnitude (*r* ^2^ = 0.19, *p* = 0.0003, *b*_0_ = 0.65). When split for age this relationship between leucine dose and rate of rise to peak plasma leucine concentration was present for both younger (Figure 4b; *r* ^2^ = 0.39, *p* < 0.0001, *b*_0_ = −1.02) and older adults (Figure 4c; *r* ^2^ = 0.16, *p* = 0.04, *b*_0_ = 2.21). However, correlations were not observed between peak plasma leucine concentrations and delta change in postprandial, postexercise MPS across the entire dataset (Figure 4d; *r* ^2^ = 0.01, *p* = 0.28, *b*_0_ = 111.3) nor when split into younger and older adults (Figure 4e; *r* ^2^ = 0.01, *p* = 0.52, *b*_0_ = 113.2, Figure 4f; *r* ^2^ = 0.05, *p* = 0.26, *b*_0_ = 95.52 for younger and older adults, respectively).

#### 3.7.3 Total postprandial plasma leucine availability

When considering the entire dataset (Figure 5a), the leucine dose showed a relationship to plasma leucine iAUC (*r* ^2^ = 0.58, *p* < 0.0001, *b*_0_ = −5701). A significant relationship was observed between leucine dose and plasma leucine iAUC for both younger (Figure 5b; *r* ^2^ = 0.74, *p* < 0.0001, *b*_0_ = −10,850) and older (Figure 5c; *r* ^2^ = 0.65, *p* < 0.0001, *b*_0_ = −1939) adults. However, plasma leucine iAUC showed no relationship to delta change in postprandial, postexercise MPS rates when considering the whole dataset (Figure 5d; *r* ^2^ = 0.02, *p* = 0.27, *b*_0_ = 124.6) and this relationship was not altered when split for age; plasma leucine iAUC showed no relationship to delta change in postexercise MPS rates among the younger (Figure 5e; *r* ^2^ = 0.002, *p* = 0.78, *b*_0_ = 131.7) or older (Figure 5f; *r* ^2^ = 0.01, *p* = 0.65, *b*_0_ = 96.62) adults.

## 4 DISCUSSION

### 4.1 Principal findings

In the present quantitative systematic review, we provide a detailed examination of the physiological regulation of postexercise MPS rates by leucine ingested within dietary protein in younger and older adults. We first quantified the predictive capacity of leucine dose per se on the magnitude of postexercise MPS rates. We then sequentially examined the relationships between ingested leucine dose and various aspects of its postprandial postexercise availability within the circulation. Finally, we assessed the ability of those aspects of postprandial plasma leucine availability to predict postexercise MPS rates. We report several novel findings that further our understanding of the leucine threshold and trigger concepts. First, ingested leucine dose per se is associated with the magnitude of the postprandial postexercise MPS response, but this relationship exists only in older adults, over both the early and entire measurement periods (Figure 2c). Second, largely irrespective of age, ingested leucine dose is highly predictive of the peak magnitude, rate of rise, and total availability of plasma leucine concentrations during the postprandial postexercise period. Finally, when examining these discreet aspects of postprandial postexercise plasma leucine variables in this reductionist manner, no single variable possessed any association with the magnitude of postprandial postexercise MPS rates in either young or older adults.

### 4.2 Leucine threshold concept

What is generally referred to as the “leucine threshold” hypothesis posits a simple dose–response relationship between total leucine ingested and the postprandial postexercise MPS response, plateauing at around ~2.5 g (Witard et al., 2014). This is aligned with various applied sports nutrition recommendations to ingest a protein meal containing at least 2–3 g leucine in close temporal proximity to exercise to maximize the postexercise muscle anabolic response (Collins et al., 2021; Dickinson et al., 2013; Katsanos et al., 2006; Morgan et al., 2022; Phillips & Van Loon, 2011; Wall, Morton, et al., 2015). Our present data do not fully support this concept. There was no correlation between ingested leucine dose and the postexercise MPS response over a 6‐h period (Figure 2) in the largest cohort of young individuals studied to date. Indeed, the lines for both the early and entire phase of postexercise MPS intercept (*b*_0_) at around 100% could already be maximal (Figure 2b). Some (Atherton et al., 2017; Dreyer et al., 2008; Gwin et al., 2021), but not all (Churchward‐Venne, Breen, et al., 2014; Fujita et al., 2009), studies demonstrate an increase in MPS with additional leucine over resistance exercise alone, which raises the question of whether leucine increases MPS over and above the stimulus of resistance exercise in younger individuals at all. Of course, there could be a dose–response effect on suppressing MPB and, therefore, hypertrophy, but this has not been investigated to date. This also highlights the difficulty in providing precise prescriptions based on leucine alone, especially given the array of differences across subjects and exercise protocols. In contrast, there was a strong dose–response correlation between ingested leucine dose and postexercise MPS rates in older adults, with overall lower increases observed compared with young. Indeed, in comparison with younger adults where *b*_0_ was around 100%, MPS did not increase to 100% (i.e., double) at all over 2 h, or until around 3–4 g of leucine was ingested during the entire postprandial phase. This is in line with recent similar investigations into the regulation of MPS by leucine in older individuals (Wall et al., 2013) and likely reflects age‐related alterations in digestion and absorption kinetics (Gorissen et al., 2020; Milan et al., 2015), splanchnic extraction (Boirie et al., 1997; Volpi et al., 1999), perfusion (Timmerman et al., 2010), and/or a reduction in sensitivity (and/or delay in response) of muscle to the anabolic properties of dietary protein (all encompassed within the term “anabolic resistance”; Burd et al., 2013; Cuthbertson et al., 2005; Wall, Gorissen, et al., 2015). In line, older adults showed a slower rate of rise to peak, greater variability of peak magnitude and an overall “rightward shift” that was lower, particularly in the early phase, indicating a greater and faster leucine response is required for an equivalent rise in MPS. To a certain extent, this shift to the right could explain the significant correlation in the older adults only, given this provides a greater spread of the data. Irrespective, our findings extend the concept of anabolic resistance to imply that the anabolic sensitivity to leucine becomes of more relevance in terms of governing postexercise postprandial MPS rates in senescent muscle. However, the linear nature of the relationship between leucine dose and postexercise MPS rates in older individuals, and lack of an obvious breakpoint, do not reveal a plateau or “threshold,” unlike previous studies that only compare two or three doses (Moore, Robinson, et al., 2009; Witard et al., 2014; Yang et al., 2012).

### 4.3 Leucine trigger concept

The utility of comparing leucine dose to postexercise MPS responses does not account for the multitude of mediating physiological factors that could mechanistically modulate this relationship. Attempts have been made to link the two, generally encompassed within the umbrella term “leucine trigger” hypothesis (Phillips & Van Loon, 2011; Witard et al., 2014; Zaromskyte et al., 2021). We show that leucine dose strongly predicts various postprandial candidate “triggers,” such as peak plasma leucine magnitude (Figure 3; Norton et al., 2009; Pennings, Boirie, et al., 2011; Tang et al., 2009; West et al., 2011), the rate of rise to peak plasma leucine magnitude (Figure 4; Burd, Yang, et al., 2012; Wall et al., 2013), and total postprandial plasma leucine availability (iAUC; Figure 5, Mitchell, Phillips, et al., 2015). However, when comparing these variables against postprandial MPS rates, no relationships were observed in the entire cohort, nor when younger and older adults were considered separately (Figures 3, 4, 5). This is surprising given the observed association between leucine dose and postexercise MPS rates, as well as the prevailing wider narrative within the literature where a clear manipulation of postprandial leucinemic variables per se and an association with the consequent muscle anabolic response are seen. West et al. (2011) reported that a more rapid delivery of leucine to the circulation following bolus whey ingestion translated to greater MPS rates compared with the same quantity consumed in a pulse fashion. Similarly, by comparing ingestion of prehydrolyzed casein with intact casein, Pennings, Boirie, et al. (2011) showed greater leucinemia conferred a more potent MPS response in older adults.

The lack of any observed associations, within this systematic review, may be explained by “noise” in the data being too great to pin down one single plasma variable, whereas the leucine dose represented a composite of the total protein dose and all postprandial leucinemic factors thereby revealing the relationship. However, once other variables are introduced, such as comparing different protein sources (Chan et al., 2019; Churchward‐Venne, Breen, et al., 2014; Dideriksen et al., 2011; Reidy et al., 2013), isolated vs whole foods (Burd et al., 2015; Mitchell, McGregor, et al., 2015; Van Vliet et al., 2017), meal ingestion (Kim et al., 2016; Symons et al., 2011) or co‐ingestion with other macronutrients (Gorissen et al., 2014; Hamer et al., 2013; Koopman et al., 2007; Staples et al., 2011), the relationship is far less clear. We (Monteyne, Coelho, Porter, Abdelrahman, Jameson, Finnigan, et al., 2020; Monteyne, Coelho, Porter, Abdelrahman, Jameson, Jackman, et al., 2020; West et al., 2022) and others (Burd et al., 2015; Chan et al., 2019; Van Vliet et al., 2017) have observed a dissociation between circulating leucine concentrations and MPS in a series of recent studies, specifically involving whole food approaches. For example, a 25% greater MPS response was observed with ingestion of skim milk vs. beef despite a significantly lower plasma leucine concentration (Burd et al., 2015). In line, a recent systematic review concluded that the leucine trigger hypothesis was predictive of subsequent MPS responses only if protein isolates were consumed on their own (Zaromskyte et al., 2021). This may also explain why we only observed a relationship between leucine threshold and MPS in older individuals, where all the studies to date have involved protein isolates.

Collectively, therefore, it appears that postprandial plasma leucine responses as the prime determinant of the postexercise MPS response may be of most relevance when all other factors remain the same, and thus, leucine availability is limiting. Once other factors are introduced the influence of leucine diminishes and other regulatory candidates and limiting factors (e.g., total protein dose, other signaling or substrate limiting amino acids, other macro/micronutrients, and hormonal/incretin/neural) must be considered. However, the total protein dose provided did not modulate delta change MPS (Figure S1—https://doi.org/10.6084/m9.figshare.22203514), and as such, any observed relationships between leucine and delta change MPS did not appear to be primarily driven simply by a greater dose of leucine also being associated with a larger dose of total protein. It is important to note that true plasma leucine kinetics, involving multiple pool modeling of exogenous and endogenous leucine rates of appearance and disappearance, as well intramuscular transport, incorporation, oxidation, and efflux, are rarely measured. One might hypothesize that protein sources “other factors” aforementioned could speed the rate of disappearance of leucine into muscle tissue for a greater intracellular stimulatory effect on MPS, while also lowering peak magnitude, rate of rise, and/or total postprandial availability of plasma leucine. Indeed, there has been much debate as to where a potential leucine “sensor” may reside (Wolfson et al., 2016), with an intracellular sensor now considered most likely (Taylor, 2014). Therefore, using plasma leucine variables (only) as proxy markers for MPS triggers may not be an effective tool. We and others may be neglecting key variables such as changes (independently from plasma concentrations) in muscle leucine uptake, intracellular leucine concentration, and intramuscular leucine incorporation into polypeptide chains.

### 4.4 Conclusions and limitations

This systematic review collated all studies, which have provided a single bolus of protein within 1 h of a single bout of resistance exercise and measured the subsequent MPS response. While there is a clear dose–response of ingested leucine with postexercise MPS rates in older individuals, our data do not identify a precise leucine *threshold*, as no evident plateau was identified, and a maximal MPS response appears to be achievable in young individuals with protein ingestion per se irrespective of leucine content. Moreover, we report that the postexercise postprandial MPS response cannot be predicted from any single plasma leucine variable and, therefore, we cannot confirm the existence (or at least primacy) of a specific physiological leucine *trigger*. As such, our results indicate that both leucine dose and plasma leucine concentrations only explain part of the variability in postexercise postprandial MPS responses. Given our data are somewhat at odds with in vitro findings and some individual studies, we leave open several possibilities that our conclusions may be obfuscated by: lack of data across more diverse leucine doses (most studies provided 2–2.5 g leucine) or corrected to total body/lean mass; few reports involving true postprandial leucine kinetics; lack of intramuscular leucine measurements; altered and/or additional regulation by (as yet unidentified) other macro/micronutrients; and the availability of other amino acids required as signal and/or substrate for sustaining optimal MPS rates. Nevertheless, this review has again highlighted anabolic resistance in older individuals and the importance of study design with older individuals needing to encompass a longer postprandial period to ensure that the whole MPS response is captured.

## AUTHOR CONTRIBUTIONS

Kiera Wilkinson, Christopher P. Koscien, Alistair J. Monteyne, Benjamin T. Wall, and Francis B. Stephens conceived and designed the study; Kiera Wilkinson, Christopher P. Koscien, and Alistair J. Monteyne conducted the search, study selection, and data extraction; Kiera Wilkinson analyzed the data; Kiera Wilkinson, Alistair J. Monteyne, Benjamin T. Wall, and Francis B. Stephens contributed to the interpretation of the data; Kiera Wilkinson prepared figures; Kiera Wilkinson, Alistair J. Monteyne, Benjamin T. Wall, and F.B.S. drafted the manuscript; Kiera Wilkinson, Benjamin T. Wall, and Francis B. Stephens edited and revised the manuscript; Kiera Wilkinson, Christopher P. Koscien, Alistair J. Monteyne, Benjamin T. Wall, and Francis B. Stephens approved final version of the manuscript.

## FUNDING INFORMATION

K.W. PhD studentship is supported by a grant from Beachbody LLC.

## CONFLICT OF INTEREST STATEMENT

No conflicts of interest, financial or otherwise, are declared by the authors.

### ETHICS APPROVAL

Not applicable.

## Supporting information


Data S1.

Data S2.

Figure S1.

## Data Availability

The datasets generated and/or analyzed during the study implementation are available from the corresponding author upon request.
