# Supplementary figures and images for: Association of postprandial postexercise muscle protein synthesis rates with dietary leucine: A systematic review
Source: Physiol Rep. 2023 Aug 3;11(15):e15775. doi: 10.14814/phy2.15775 (PMC10400406; doi:10.14814/phy2.15775)

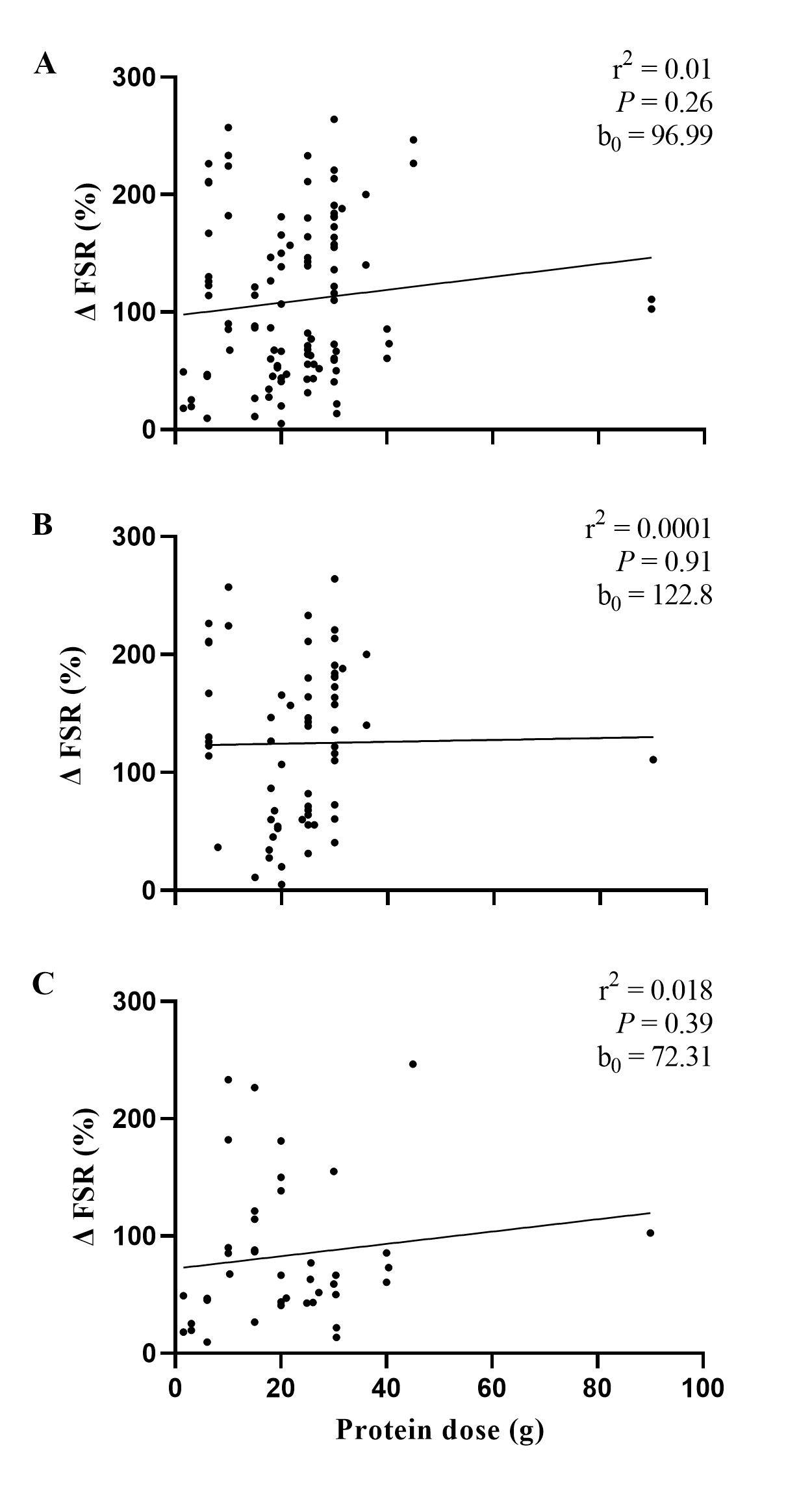

Supplement: Supplementary file 3 — Figure S1. [file PHY2-11-e15775-s002.tif]
